# Testing Various Cement Formulations under Temperature Cycles and Drying Shrinkage for Low-Temperature Geothermal Wells

**DOI:** 10.3390/ma16237281

**Published:** 2023-11-23

**Authors:** Hartmut R. Fischer, Al Moghadam

**Affiliations:** 1 TNO Materials Solutions, High Tech Campus 25, 5656 AE Eindhoven, The Netherlands; 2 TNO Applied Geosciences, Princetonlaan 6, 3584 CB Utrecht, The Netherlands; al.moghadam@tno.nl

**Keywords:** cement, geothermal, drying shrinkage, fibers, rubber, additives, temperature cycles, radial cracks, leakage, microannulus

## Abstract

Low-enthalpy geothermal wells are considered a sustainable energy source, particularly for district heating in the Netherlands. The cement sheath in these wells experiences thermal cycles. The stability of cement recipes under such conditions is not well understood. In this work, thermal cycling experiments for intermediate- and low-temperature geothermal well cements have been conducted. The samples were cured either under ambient conditions or under realistic pressure and temperature for 7 days. The samples did not show any signs of failure after performing 10 cycles of thermal treatment between 100 °C and 18 °C. We also tested cement formulations under drying conditions. Drying shrinkage is caused by a reduction in the water content of cement, which leads to capillary forces that can damage cement. Such circumstances lead to tensile stresses causing radial cracks. Most samples exhibited cracks under low humidity conditions (drying). Fiber reinforcement, especially using short PP fibers, improved the cement’s resilience to temperature and humidity changes. Such additives can improve the longevity of cement sheaths in geothermal wells.

## 1. Introduction

In order to meet the targets set in the Paris Agreement, fossil fuels need to be replaced with other sources of energy. Consequently, the supply of energy in the future is anticipated to come from a mix of various renewable sources, including geothermal energy. This particular energy source can provide significant amounts of both electrical and thermal energy, up to, 3% and 5%, respectively, of the global demand in 2050 [1]. However, geothermal wells must be durable and sustainable to reduce the financial risks, thereby making investment in geo-energy more attractive. 

Geothermal pipes are susceptible to corrosion (rust). Produced water from geothermal wells has high temperatures and high salinity and contains chemicals (some naturally occurring, and others are added to prevent corrosion) [2,3]. If corrosion weakens or generates a hole in the pipes, salt water can leak into the freshwater aquifers that provide drinking water. Such integrity issues in wells are costly to repair and jeopardize the economics of geothermal projects. 

The current technology for sealing is the placement of well cement into the annulus between the casing and formation to provide mechanical stability and structural support as well as zonal isolation. Well leakage can occur due to the failure of the cement interfaces in the well, either the cement/casing interface or the cement/formation interface [4,5]; leakage along the cement interfaces can expose the casing to corrosive fluids. Consequently, attention needs to be paid to the behavior of cement under challenging geothermal conditions such as (high) temperature variations [6]. These conditions subject casing and cement sheaths to thermal and stress shocks as well as chemically aggressive environments. Thermal cycling due to a pause and resumption of activity in geothermal wells causes casing, cement, and formation to repeatedly expand and contract according to their distinct thermal expansion coefficients, thereby introducing stresses on and between the wellbore components [6]. As a result, failures such as debonding, shear, and radial cracking of the annular cement sheath can occur [7,8]. Both the amplitude and the frequency of the temperature cycles can initiate damage to the cement seal, and cyclic loading can lead to fatigue growth of defects/failures within the system, compromising zonal isolation, which may result in casing corrosion and deterioration of well integrity. The cycles may be of short (daily) or long (seasonal) frequencies. Even for intermediate- and low-temperature wells, common Ordinary Portland Cement (OPC)-based formulations may develop problems with strong temperature variations [4,6]. 

Consequently, a cement recipe generating a material resistant to thermal cycles is required for the robust long-term performance of a geothermal installation. Such resistance can be achieved by either increasing the strength of the cement to withstand the forces generated during temperature cycling by designing harder and stiffer cement formulations, e.g., by using silica fume as an additive or applying flexible cements, which are more able to withstand the stresses generated by fluctuations in downhole pressure and temperature. An introduction of micro-fibers such as wollastonite [9] or other fibrous materials [10] results in an increase in flexural strength and the promotion of pore discontinuity/permeability reduction in cement. Furthermore, fiber reinforcement increases the resistance to cracking induced by thermal effects or shrinkage by arresting the crack growth and by transferring stresses across a crack [8]. A review by Doğan and Demir [11] discusses all aspects of fiber-reinforced concrete with a comparison of concrete properties, such as shrinkage and crack formation, compression, splitting tensile and flexural strength, toughness, and elastic modulus. As a result, it was shown that the addition of (polymer) fibers increases cement durability [8].

Improved cement ductility can also be achieved by introducing tire rubber particles into cement-based materials [12]. Tire rubber particles have the potential to enhance the toughness of cement by interfering with crack propagation processes through the dissipation of stresses. The ductile deformation of the rubber particles leads to the stabilization of defects and the prevention of brittle cracking [13]. 

Additionally, polymer latex is widely used in civil engineering applications for an improvement in the service performance of concrete and mortar [14]. The use of a ductile polymer as a modifier seems to be a promising strategy for improving microstructure and enhancing the durability of cement mortar and concrete [15]. Styrene butadiene rubber (SBR) latex has been widely used in fresh mortar and concrete [16]. In the case of mortars modified by latex rubbers, Justnes and Øye [17] showed that the latex forms a continuous network if the polymer content exceeds 10 wt.%. This polymer network explains the improvement in the tensile and/or flexural strength. Song et al. used a combination of latex powder and rubber to modify oil in well cement recipes [18]. This combination has shown a significant improvement in flexural strength, impact strength, and the ductility of the cement as well as in decreasing fluid loss of the slurry. A mixture of 3 wt.% latex powder and 2 wt.% rubber results in the formation of a three-dimensional, flexible network structure, which improves the elasticity and toughness of cement stone.

The aim of this study is to conduct a first-order investigation of suitable Portland cement recipes that can withstand low-enthalpy geothermal well conditions. Based on the literature review, appropriate additives that may improve cement’s ductility and strength have been identified (rubber particles, latex, fibers, silica fume, etc.). The recipes are tested by exposing them to temperature cycles and low humidity conditions (drying). A qualitative analysis of the cement damage, i.e., cracking, has been conducted to identify promising cement recipes for future experiments. The results of this study identify promising cement recipes for further testing, in order to find improved cement recipes for geothermal wells. 

## 2. Materials and Methods

### 2.1. Cement Formulations

Various formulations were made by mixing different ingredients, including rubber particles, silica fume, latex, and polymer fibers. All the recipes tested included class G cement (Dyckerhoff Well Cement) as the base material. The additives tested in this work are as follows:

- Crumb rubber powder from waste tire rubber (WTR) with a granule size of less than 0.5 mm obtained from Kargo Recycling Nederweert, The Netherlands;
- Basoblock™ LD 105 (Ludwigshafen, Germany), a styrene–butadiene latex from BASF;
- Paragas^®^, a modified polyethylenimine (water-soluble polymer) from BASF;
- Polypropylene (PP) and polyacrylonitrile (PAN) fibers 3 mm in length and 35 and 12micron in diameter from Shandong Dachuan New Materials Co., Ltd., Taian, China;

An overview of the samples prepared is given in Table 1.

The formulations in Table 1 were dry blended. Cement slurries were prepared by adding 44% bwoc of water using a stirrer at 3000 rpm. The slurry was poured into the mold after 3 min of stirring. 

### 2.2. Sample Preparation

**Figure 1:**
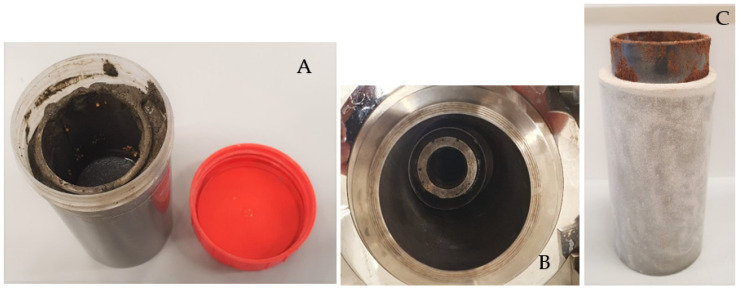
(**A**) Sample preparation with a steel pipe in the PP mold; (**B**) sample placed in an autoclave; (**C**) de-molded sample after 7 days of curing.

Samples with different geometry were prepared depending on the test. Down-scaled annular samples were designed to represent downhole cement sheaths in geothermal wells. The samples were created by placing steel pipes with an inner diameter of 40 mm and a wall thickness of 2 mm in polypropylene (PP) molds with an inner diameter of 60 mm. The length of the samples was 100 mm. The gap between the steel pipe and the PP mold was initially filled with seawater or freshwater. Seawater was used for some samples to investigate the impact of salinity on the results since most of the wells contain saline (sea) water during cementation. The cement slurry was then poured into the gap replacing seawater (Figure 1A). Subsequently, the mold was closed and allowed to cure under ambient conditions for 7 days before the mold was removed (Figure 1C). 

**Figure 2:**
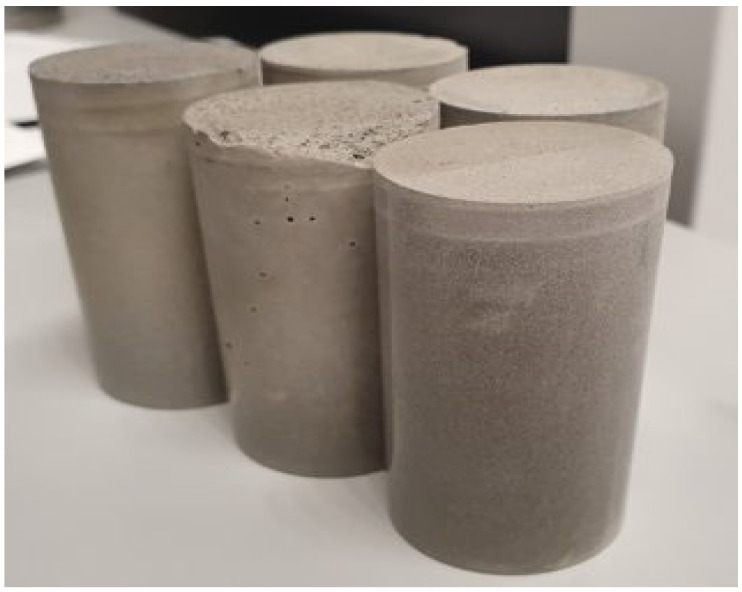
Core samples of different cement formulations to validate crack development under drying shrinkage.

Additionally, core samples to test the drying shrinkage of cores were prepared by pouring the formulations prepared as described above in cylindrical PP molds with an inner diameter of 30 mm and a height of 65 mm (Figure 2). After curing for 7 days under ambient conditions, the samples were de-molded and aged under various relative humidity conditions for 1 month.

### 2.3. Thermal Cycling Tests

For thermal testing, the annular samples (still in plastic molds) were placed in 1 L autoclaves and cured for 7 days at 100 °C and 100 bar pressure (Figure 1B). The molds were inserted in seawater with the water level below the top of the PP mold to supply moisture during curing and to avoid the leaching of cement components. Thermal cycling tests were performed by applying moderate thermal cycles (100 °C → 18 °C → 100 °C) with a heating speed of 5 K/min and cooling speed (quenching using ice-water) of 20 K/min. Each sample was exposed to 10 thermal cycles, as described. The impact of confining stress on cement was investigated in these experiments.

After the thermal cycling, the samples were inspected for damage (cracks) using optical and acoustic scanning microscopy (SAM) and a Sonix Echo with a 30 MHz acoustic lens in reflection mode. Selected samples were also analyzed via CT scanning (Phillips, Amsterdam, The Netherlands) as well as via scanning electron microscopy (SEM).

### 2.4. Drying Shrinkage Tests

**Figure 3:**
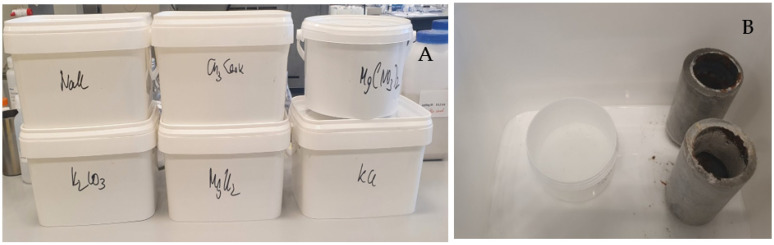
(**A**) Close boxes to store samples under defined and controlled RH; (**B**) samples inside of the box showing saturated salt solution for defining a controlled RH.

A selection of cement plugs and all annular samples (after 7 days of curing) were exposed to different constant relative humidity (RH) environments in closed boxes to study the effect of drying shrinkage. RH values were controlled by having saturated salt solutions inside the closed boxes (see Figure 3, Table 2). The salt solutions in Table 2 were selected to cover a wide range of RH [19]. The samples stored under conditions of constant RH were inspected daily for damage, and the width of appearing cracks was measured using a Keyence digital microscope VHX 600 (Keyence corporation, Osaka, Japan). Furthermore, the hardness of the cement formulations was measured using a microindenter Fischerscope H100C (Helmut Fischer Holding GmbH, Sindelfingen, Germany). 

## 3. Results

### 3.1. Thermal Cycling Tests

**Figure 4:**
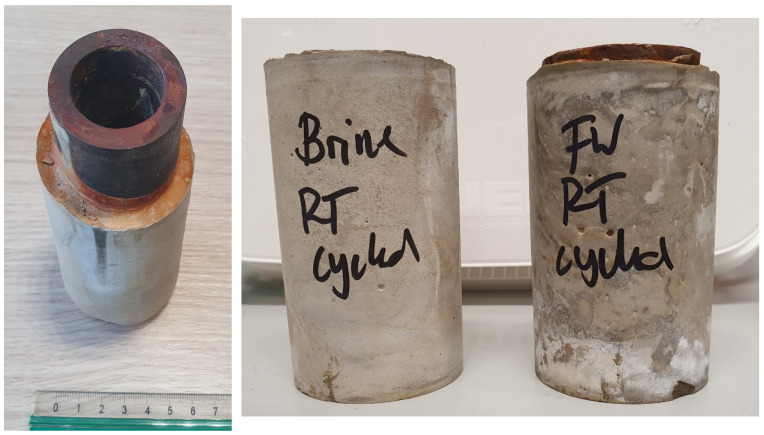
Samples after 10 cycles of temperature cycling as retrieved from the autoclaves.

A selection of samples listed in Table 1 were exposed to thermal cycling at 100 °C and 100 bar pressure. All the tested samples showed no sign of damage after 10 cycles between 100 and 18 °C, using optical and acoustic microscopy. The curing pressure and temperature and the displaced fluid (freshwater versus saltwater) did not change the results. Figure 4 shows an image of cement samples after the cycling test. Visually, the cement that displaced saltwater in the annulus looked different, but optical microscopy indicated no damage in the samples. The results show that in low-enthalpy geothermal wells, temperature cycles alone do not necessarily damage class G cement. However, in the tests performed, the stress conditions in cement are not completely the same as those present under downhole conditions [20]. Therefore, it does not rule out the impact of thermal stresses on cement. 

### 3.2. Drying Shrinkage Tests

**Figure 5:**
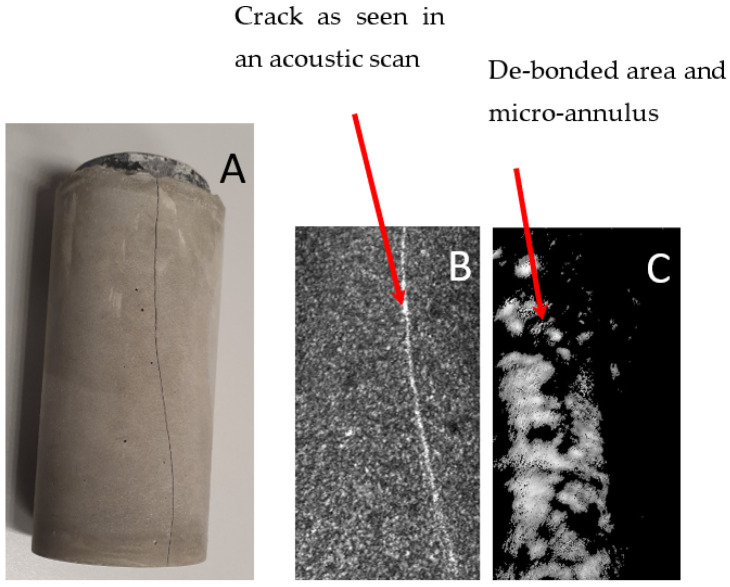
(**A**) Sample TestCEM B after thermal cycling and ageing in air showing radial cracking; (**B**) acoustic scan beneath the outer surface of the cracked sample TestCEM B; and (**C**) acoustic scan of the same sample at the cement–steel interface showing partial de-bonding and the formation of a micro-annulus.

**Figure 6:**
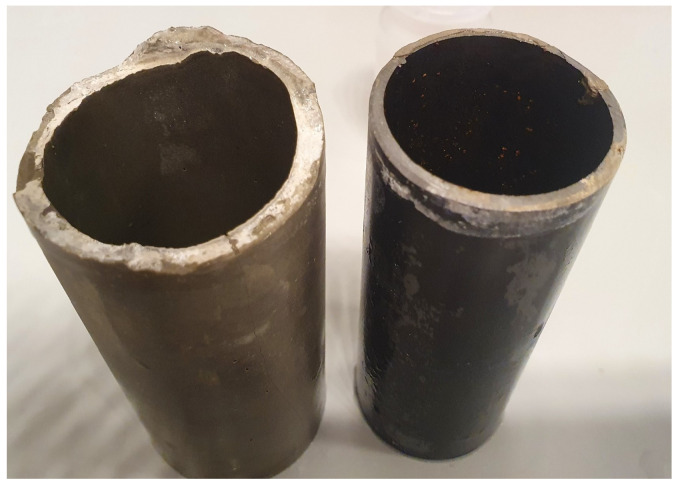
TestCEM B sample after thermal cycling and ageing in air after total debonding of the cement–steel interface showing complete separation of cement and steel pipes without destruction of the cement part.

Samples aged/stored in air eventually developed tensile cracks as shown in an acoustic scan in Figure 5A. These cracks appeared after approximately two weeks of storage under ambient conditions (55% RH, 23 °C). An investigation via scanning acoustic microscopy indicated the extension of the cracks based on the whole thickness/length of the cement sheath (Figure 5B). The tensile tension initiated crack partially coincided with the debonded area of the cement sheath from the steel pipe as detected via SAM (Figure 5C). De-bonding was seen on the left side as a micro-annulus filled with air, which caused a bright contrast. The de-bonded cement sheath and the steel pipe could be easily separated from the cement sheath pipe (Figure 6).

**Figure 7:**
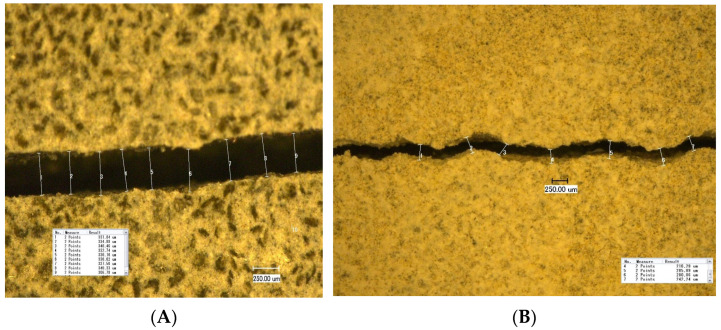
Crack openings of the samples TestCEM B (**A**) and TestCEM si35 (**B**) after storage under 43% RH.

This indicates that cement integrity is more sensitive to drying than temperature cycles alone. Drying in cement causes partial desaturation of pore fluids, which in turn induces capillary stress in the sample. This leads to drying shrinkage that can cause cracks, which is an effect known and intensively studied for concrete [21,22,23,24,25]. Consequently, cured samples were exposed to different RH levels (Table 2) to investigate the resistance of each formulation to drying shrinkage as an overall measure of ductility and tendency for brittle cracking. All formulations were exposed to RH levels between 22.7 and 84%. The appearance of crack(s), mostly radial, was recorded over time, and the width of the crack opening was measured using a digital microscope. At least three different positions of the crack and at least six measurements per position were performed (Figure 7).

**Figure 8:**
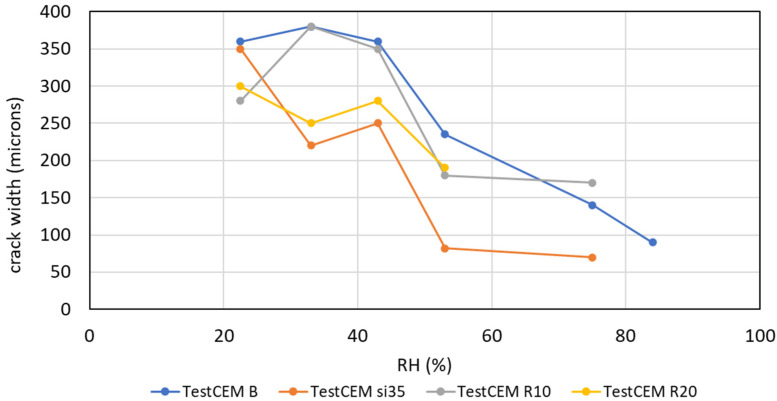
Crack opening (width) depending on storage RH.

It was found that the developed crack width (opening) depends on the RH value; storage under conditions with lower RH results in the development of a larger crack width. Figure 8 shows the dependency of the crack opening on the RH of the storage for four formulations. TestCEM B sample (neat class G) shows an initial crack at 84% RH with an aperture of approximately 90 microns. As humidity decreases, the size of the aperture increases to 350 microns at 43% RH. The aperture remains constant as RH decreases below 43%. The TestCEM Si35 sample shows an initial crack at 75% RH with an estimated aperture of 60 microns. The size of the crack increases at lower RH levels to 350 microns. TestCEM R10 sample shows initial cracking at 75% humidity starting at 170 microns. The TestCEM R20 (20% rubber particles) shows the most resistance to cracking, where the first crack appears at a humidity of 53%. 

**Figure 9:**
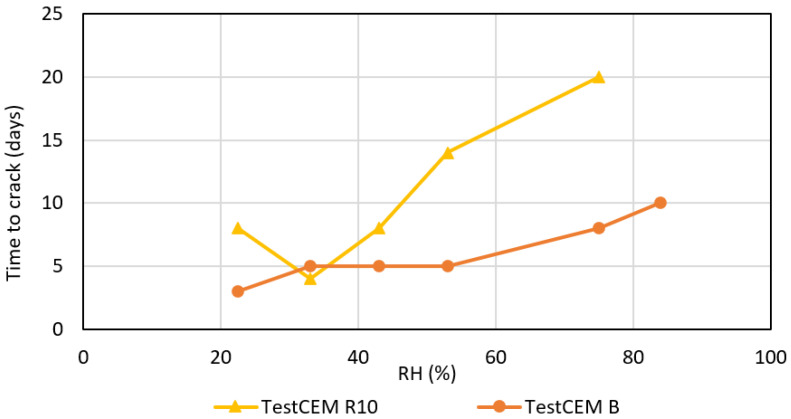
Crack opening (width) time to crack appearance depending on storage RH.

**Figure 10:**
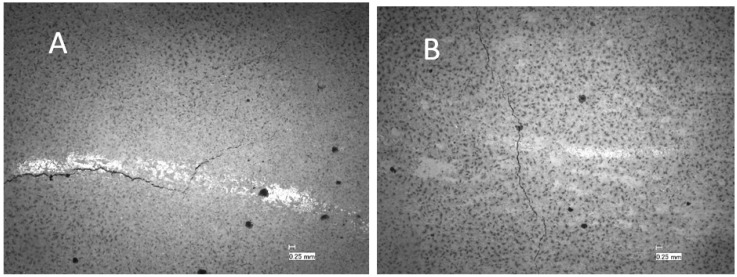
Crack openings of the samples TestCEM PP (**A**) and TestCEM PAN-R10 (**B**) after storage under 43% RH.

Figure 9 shows the time it takes for the crack to appear at different humidity levels. TestCEM B sample shows cracking after 10 days at 83% humidity. However, the TestCEM R10 takes 20 days to show the first crack at 75% humidity. These results indicate that the samples including rubber take longer to develop cracks at higher RH values. At RH below 40%, both samples show cracks at the same time frame. Samples TestCEM PP, TestCEM PAN, TestCEM PAN-R10, and TestCEM PAN BB only show small, non-continuous cracks (15–30 microns, see Figure 10) even at RH less than 43%. A precise measurement of the crack width is somewhat difficult in case of very large openings since such samples also show partial delamination/debonding of the cement sheath with the steel pipe and, therefore, need additional care during the measurements. Also, the samples show a (partial) closure of the cracks if stored (again) in a higher RH environment after crack formation. 

**Figure 11:**
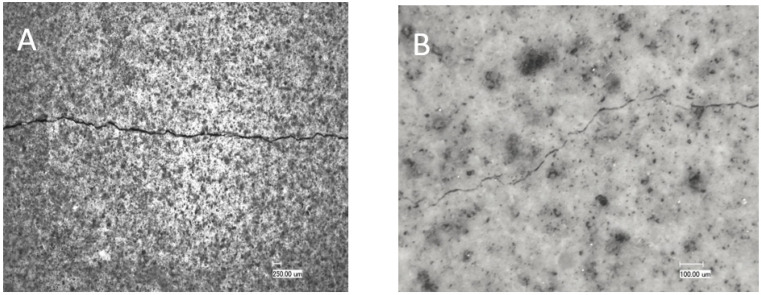
Microscopic pictures of the cracks developed in core samples of TestCEM B (**A**) and TestCEM PG (**B**).

Core samples also developed cracks after drying due to shrinkage. However, due to the geometry of these samples compared to annular sheath samples, these cracks were not as wide as the cracks observed at the cement sheaths. The core samples exposed to low-humidity conditions, unmodified class G (TestCEM B), and silica-modified samples (TestCEM si35) showed the widest crack openings at 45 microns (Figure 11A). Ductile formulations such as 10% rubber particle-modified samples (TestCEM R10) showed slightly smaller cracks at 35 microns. The 20% latex-modified samples (TestCEM PG) indicate the smallest crack widths at an average of 12 microns. Figure 11B shows only minor cracking or no cracking (Table 3). A summary of the results for all cement recipes is provided in Table 4.

## 4. Discussion

### 4.1. Thermal Cycling

Thermal cycling in intermediate- and low-temperature geothermal wells causes the formation, cement, and casing to expend and contract frequently following the specific thermal expansion coefficients. This results in stress, depending on the mechanical interaction/contact of the different components, and possibly failure such as debonding, radial, and shear cracking of the annular cement sheath. Exposing the cement recipes considered in this work to moderate temperature cycles did not induce any cracks. The observation was similar for all the recipes. This indicates that the temperature cycles alone do not cause cracks in cement for the range of temperatures tested in this study. Therefore, the focus should be on the mechanical properties of cement, e.g., ductility, to ensure that cement recipes can withstand geothermal conditions. It should be noted that in this work the impact of thermal stresses was not investigated. 

### 4.2. Impact of RH

In cases where an external source of water is not present, cement hydration can cause drying under in situ conditions [20]. This can occur when cement is placed against highly impermeable rocks, such as caprock shales or salt formations. This may lead to a short-term reduction in the relative humidity of the cement sheath. The results in this work show that at low relative humidity, class G cement is prone to cracking. However, some of the formulations tested in this work, namely TestCEM PP, TestCEM PAN, TestCEM PAN-R10, and TestCEM PAN BB, showed significant resilience in withstanding dry conditions. 

Any significant change in the RH of the environment during the cement preparation, curing, and testing process significantly affects the size of the gap at the cement/casing interface in the annular cement samples. The gap size between the cement and the casing is inversely proportional to the change in the RH of the environment [24]. This behavior has been observed in the experiments in this work; depending on the RH of the storage, damage by radial cracking occurred and developed over time. Ductile formulations containing latex, rubber particles, and a combination thereof performed somewhat better while developing cracks later in time. However, the developed crack width was the same for all formulations, regardless of whether they were ductile or made harder by adding silica fume (particle size ca 400 μm). The only exceptions were fiber-reinforced formulations, where the crack width was limited (ca 60 μm) and the cracks that developed were non-continuous and isolated. 

### 4.3. Impact of Latex

A synergistic effect of latex powder and rubber on the properties of oil well cement-based composites has already been described earlier by Song et al. [18]. In this combination, a synergism between latex powder and rubber led to the formation of a three-dimensional network structure and a flexible structure, which subsequently improved the elasticity and toughness of cement. The improved elastic matrix had a buffering effect on the external impact when the cement was subjected to an external load. However, this effect was not observable in the experiments described in this study. A synergistic effect has not been noticed or was not large enough to cause a substantial improvement in the drying shrinkage resistance of the tested formulations. An improvement compared to class G cement was noticed while adding both components; however, the formulations still developed cracks although with smaller dimensions/openings. 

**Figure 12:**
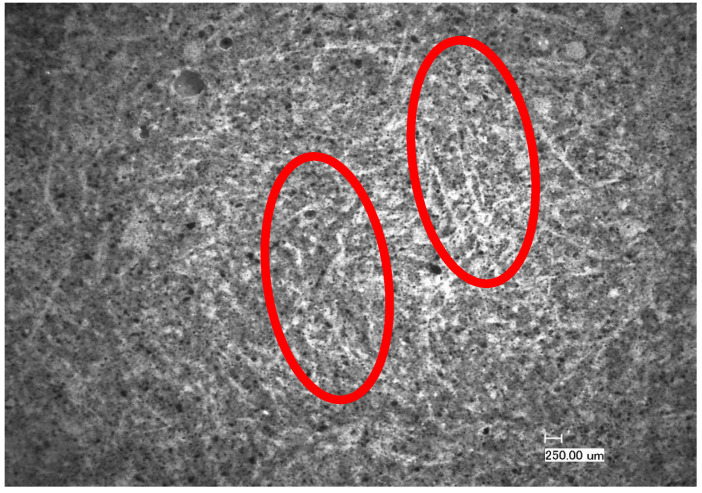
Microscopic picture of the morphology of the sample TestCEM PAN-BB showing the finely distributed PAN fibers embedded in the polymer latex film. Examples of the embedded fibers can be seen in the red-circled areas.

The sample TestCEM PAN-BB showed an interesting morphology, as presented in Figure 12. In this sample, the latex forms a film and facilitates the embedding and dispersion of the PAN fibers. The dispersed fibers act as reinforcing elements and the latex film enhances the toughness/flexibility of the cement. This can explain the minor cracking caused by drying shrinkage.

### 4.4. Impact of Fibers

The TestCEM PAN sample cured under ambient conditions only showed small, not continuous crack openings even at the lowest tested RH (33%). However, curing under other well conditions (100 °C and 100 bar) caused a complete thermal degradation of the PAN fibers due to hydrolysis. Consequently, PAN fiber-reinforced cement is not suitable for application in geothermal wells. In contrast, the PP fiber-reinforced sample (TestCEM PP) was able to withstand the curing and thermal cycling conditions of the well. The storage of the sample under reduced RH (33%) did not result in the development of any damage after a storage period of two months. Also, a further reduction in the PP fiber content from 1.5% to 0.45% bwoc showed the same result, with no damage development after curing under pressure/temperature and temperature cycling and 1 month of storage at 33% RH. 

**Figure 13:**
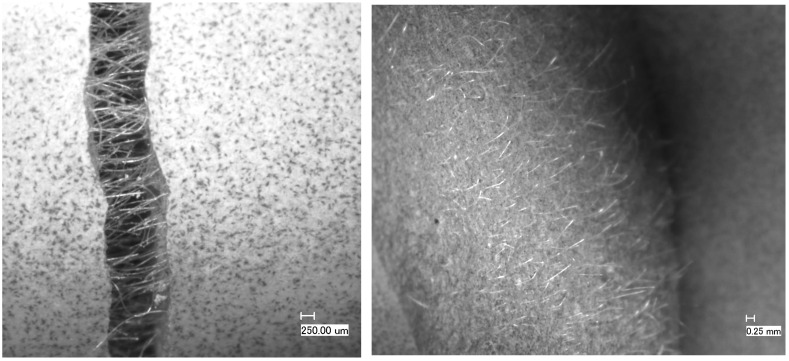
Crack-bridging effect and fracture surface of a TestCEM formulation—core sample containing only 0.15% bwoc PP fibers after 3-point bending leading to fracture. Clearly, the fibers act like they are intended to bridge the crack. The distribution of the fibers is very homogeneous, thereby ensuring the envisaged effect with a very small amount of fibers added.

The addition of fiber to cement greatly improved the tolerance to drying shrinkage, as already reported earlier [8]. The presence of fibers in the cement slurry significantly delayed cracking and restricted the crack dimensions of the radial cracks due to the fibers bridging them as well as caused a substantial reduction in the inner micro-annulus compared to specimens without fibers, thereby reducing potential leak paths. The crack-bridging effect and the distribution of the fibers in the cement matrix have been studied on fractured sample surfaces (Figure 13). The distribution of the fibers is homogenous throughout the sample, resulting in a hairy fracture surface. We did not observe any evidence of poor fiber distribution in our samples. Neutron radiography could confirm this as the single fibers were close to the resolution limit of the instrument and were difficult to see. However, bundles as-supplied in the range of up to 1 micron diameter could not be detected. 

**Figure 14:**
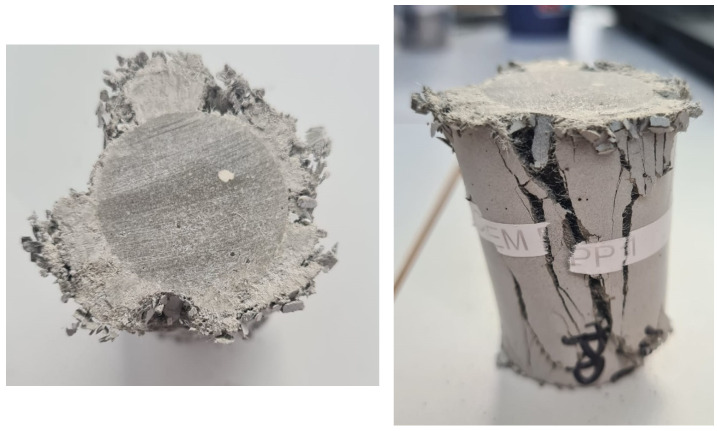
Top and side view of the TestCEM PP core sample after compression strength testing. Again, the crack-bridging effect is very clearly observable, which prevented the sample from complete disintegration and spalling.

Crack-bridging could prevent cement from catastrophic failure and complete disintegration as observed during the compression testing of core samples (Figure 14).

The addition of microfibers to mortars has shown a decrease in the drying shrinkage of 35% and 65% with an addition of 1% PAN fibers and 0.1% PAN micro-fibers, respectively [5,26]. Drying shrinkage has a direct relation with the amount of free water and the porosity of cementitious materials. This response occurs after the evaporation of the free water stored in the capillary pores due to a low environmental relative humidity. PAN (micro) fibers delayed the evaporation of the free and absorbed water, favoring the hydration of cement. Consequently, the use of PAN (micro) fibers increased the mechanical strength (compressive and flexural) and considerably decreased the drying shrinkage of the mortar. The same was observed for adding PP fibers: the shrinkage contraction rate of concrete significantly reduced after adding PP fibers [27]. This could be confirmed in this study while maintaining very low RH environments for the samples after high-temperature/high-pressure curing and thermal cycling. The use of a small amount of PP fibers is sufficient to show a high tolerance to drying shrinkage in low humidity conditions. More experiments are required to assess the ultimate strength of PP fiber-reinforced cement and its pumpability. 

## 5. Conclusions

In this study, we formulated several cement recipes that could withstand conditions relevant to low-enthalpy geothermal wells. All samples showed sufficient tolerance to temperature cycles between 18 and 100 °C. However, at low humidity, almost all samples showed signs of cracking. This was amplified in formulations with higher stiffness levels. The addition of fibers to the cement recipes has been shown to improve the microstructure of the cement and its susceptibility to drying cracks. Polyacrylonitrile (PAN) fibers disintegrate at higher temperatures and, therefore, are not a suitable candidate for geothermal wells. However, polypropylene (PP) fibers have shown great potential in improving cement’s mechanical integrity at medium temperatures, even at low concentrations. More tests are required to assess the strength and pumpability of PP fiber-reinforced cement as a candidate for geothermal wells. 

## Figures and Tables

**Table 1:** Overview of the prepared and tested samples (BWOC).

Sample	Composition	Thermal Cycling Tests	Drying Shrinkage Tests
TestCEM B	class G	Annular sample	Plug sample; annular sample
TestCEM R10	class G + 10% rubber particles		Plug sample; annular sample
TestCEM R20	class G + 20% rubber particle	Annular sample	Plug sample; annular sample
TestCEM si35	class G + 35% silica fume	Annular sample	Plug sample; annular sample
TestCEM si40	class G + 40% silica fume	Annular sample	Plug sample; annular sample
TestCEM BB	class G + 10% Basoblock		Plug sample; annular sample
TestCEM PG	class G + 20% Paragas		Plug sample; annular sample
TestCEM PP	class G + 1.5% PP fibers	Annular sample	Plug sample; annular sample
TestCEM PAN	class G + 1.5% PAN fibers	Annular sample	Plug sample; annular sample
TestCEM PAN-BB	class G + 1% PAN fibers + 10% Basoblock		Plug sample; annular sample
TestCEM PAN-R10	Class G + 1% PAN fibers +10% rubber particles		Plug sample; annular sample

**Table 2:** Salts used for the preparation of saturated solutions with a defined RH environment [19].

Saturated Salt Solution	RH (%)
CH_3_COOK	22.5
MgCl_2_	33
K_2_CO_3_	43
Mg(NO_3_)_2_	53
NaCl	75
KCl	84

**Table 3:** Crack openings developed in core samples.

Sample	Crack Width Opening (μm)
TestCEM B	45
TestCEM si35	44
TestCEM R10	35
TestCEM R20	-
TestCEM PG	12
TestCEM PP	-
TestCEM PAN	-
TestCEM PAN-BB	-
TestCEM PAN-R10	-

**Table 4:** Summary of the results for each recipe.

Sample	Composition	Thermal Cycling Tests	Drying Shrinkage Tests
TestCEM B	class G	No damage	Significant cracking
TestCEM R10	class G + 10% rubber particles		Significant cracking
TestCEM R20	class G + 20% rubber particle	No damage	Significant cracking
TestCEM si35	class G + 35% silica fume	No damage	Significant cracking
TestCEM si40	class G + 40% silica fume	No damage	
TestCEM BB	class G + 10% Basoblock		Significant cracking
TestCEM PG	class G + 20% Paragas		Small non-continuous cracks
TestCEM PP	class G + 1.5% PP fibers	No damage	No damage; stable at high temperature
TestCEM PAN	class G + 1.5% PAN fibers	No damage	Small non-continuous cracks; disintegration at high temperature
TestCEM PAN-BB	class G + 1% PAN fibers + 10% Basoblock		Small non-continuous cracks; disintegration at high temperature
TestCEM PAN-R10	Class G + 1% PAN fibers +10% rubber particles		Small non-continuous cracks; disintegration at high temperature

## Data Availability

The data presented in this study are available on request from the corresponding author.
